# Autophagic flux modulation by Wnt/β-catenin pathway inhibition in hepatocellular carcinoma

**DOI:** 10.1371/journal.pone.0212538

**Published:** 2019-02-22

**Authors:** Lilia Turcios, Eduardo Chacon, Catherine Garcia, Pedro Eman, Virgilius Cornea, Jieyun Jiang, Brett Spear, Chunming Liu, David S. Watt, Francesc Marti, Roberto Gedaly

**Affiliations:** 1 Department of Surgery, Transplant Center, College of Medicine, University of Kentucky, Lexington, Kentucky, United States of America; 2 Lucille Parker Markey Cancer Center, University of Kentucky, Lexington, Kentucky, United States of America; 3 Department of Microbiology, Immunology & Molecular Genetics, College of Medicine, University of Kentucky, Lexington, Kentucky, United States of America; 4 Department of Molecular and Cellular Biochemistry, College of Medicine, University of Kentucky, Lexington, Kentucky, United States of America; 5 Center for Pharmaceutical Research and Innovation, College of Pharmacy, University of Kentucky, Lexington, Kentucky, United States of America; National Cancer Center, JAPAN

## Abstract

Autophagy targets cellular components for lysosomal-dependent degradation in which the products of degradation may be recycled for protein synthesis and utilized for energy production. Autophagy also plays a critical role in cell homeostasis and the regulation of many physiological and pathological processes and prompts this investigation of new agents to effect abnormal autophagy in hepatocellular carcinoma (HCC). 2,5-Dichloro-*N*-(2-methyl-4-nitrophenyl) benzenesulfonamide (FH535) is a synthetic inhibitor of the Wnt/β-catenin pathway that exhibits anti-proliferative and anti-angiogenic effects on different types of cancer cells. The combination of FH535 with sorafenib promotes a synergistic inhibition of HCC and liver cancer stem cell proliferation, mediated in part by the simultaneous disruption of mitochondrial respiration and glycolysis. We demonstrated that FH535 decreased HCC tumor progression in a mouse xenograft model. For the first time, we showed the inhibitory effect of an FH535 derivative, FH535-N, alone and in combination with sorafenib on HCC cell proliferation. Our study revealed the contributing effect of Wnt/β-catenin pathway inhibition by FH535 and its derivative (FH535-N) through disruption of the autophagic flux in HCC cells.

## Introduction

Hepatocellular carcinoma (HCC) is the most prevalent, primary malignancy of the liver and one of the leading causes of cancer-related deaths. Current statistics indicate this cancer affects over 700,000 people worldwide and causes an estimated 600,000 deaths annually [1, 2]. Despite improvements in prevention, early diagnosis and new treatments, the mortality of patients with HCC continues to rise and, over the past two decades, the incidence of HCC in the United States has tripled [3]. The poor prognosis for patients with HCC reflects a pattern of the initial, undetected subclinical progression that ultimately results in late diagnosis when treatment options are limited [4]. Current efforts are underway to find improved therapeutic strategies for HCC-related signaling pathways involved in the initiation and progression of tumors. Among these pathways, HCC displays altered Wnt/β-catenin signaling [5, 6] in which more than one-third of HCC cases exhibit cytoplasmic and/or nuclear accumulation of β-catenin, a finding that correlates with poor differentiation and prognosis [7]. This pathway also contributes to the maintenance of tumor initiating cells, drug resistance and metastasis [8–10]. There is increasing evidence for the interplay between the Wnt/β-catenin pathway and autophagy in different cancers [11–14]. Autophagy is a highly conserved process that targets cellular components for lysosomal-dependent degradation in which the products of degradation may be recycled for protein synthesis and utilized for energy production [15]. Autophagy also prevents accumulation of non-functional protein aggregates and organelles that are potentially damaging to the cell, and under stress-induced conditions might trigger tumor initiation [16, 17]. The fact that overactive autophagy can also support tumor development [18] underscores the important role and tight regulatory requirements of this process in normal cell development and function. In this context, targeting the autophagy pathway emerges as a novel therapeutic opportunity for cancer treatment even if the regulation of this complex process and the involvement of different cell signaling pathways remain poorly understood. In HCC, a multi-kinase inhibitor, sorafenib, which is the standard treatment for advanced HCC, reportedly enhances autophagy [19]. Chloroquine (CQ), an autophagy inhibitor, sensitizes HCC cells to the antineoplastic effects of sorafenib. This finding again suggests that modulation of autophagy represents a potential therapeutic target for HCC [18–20].

2,5-Dichloro-*N*-(2-methyl-4-nitrophenyl)benzenesulfonamide (FH535) is a synthetic inhibitor of the Wnt/β-catenin pathway that exhibits anti-proliferative and anti-angiogenic effects on different types of cancer cells [21, 22]. In previous studies, the combination of FH535 with sorafenib promoted a synergistic inhibition of HCC and liver cancer stem cell proliferation, mediated in part by the simultaneous disruption of mitochondrial respiration and glycolysis [23, 24]. In this study, we investigate the effect of FH535 on HCC tumor progression in a mouse xenograft model and explore the underlying mechanism of FH535 and its derivatives in modulating the Wnt/β-catenin-dependent autophagic flux in HCC.

## Materials and methods

### Cell lines

The HCC cell line Huh7 [25] was a gift from Dr. Guangxiang Luo (University of Alabama, Birmingham). The HCC cell lines Hep3B and PLC were purchased from American Type Culture Collection (ATCC; Manassas, VA, USA). These cell lines were cultured in Dulbecco’s Modified Eagles Medium (DMEM; Gibco, USA) supplemented with 10% fetal bovine serum (FBS; Gibco, USA), non-essential aminoacids (NEAA; Gibco, USA) and penicillin/streptomycin (Gibco, USA) and maintained in a NuAire incubator (Plymouth, MI, USA) at 37°C with 5% CO2. [26].

### Animal xenograft model

All animal experiments were performed in accord with the guidelines, rules and recommendations and was approved by the University of Kentucky Institutional Animal Care and Use Committee after approval (IACUC). Three- to four-weeks old female athymic nude mice (nu/nu, The Jackson Laboratory, USA) were housed in a pathogen-free environment at the Division of Laboratory Animal Resources of the Chandler Medical Center, University of Kentucky. To generate *in vivo* tumors, Huh7 culture cells in mid-log phase growth were collected and re-suspended in a 50% mixture of Matrigel (BD Biosciences, USA) in serum-free medium to a final concentration of 6x10^7^ cells per mL. A volume of 0.1 mL of the cell suspension was injected subcutaneously in the right flank of each mouse. Mice were weighed and checked for tumor growth every other day. When tumors reached a volume of 100 mm^3^, mice were randomly divided into two groups of 5: vehicle control group and FH535 group (receiving 15 mg of FH535/kg/day from a stock prepared in dimethyl sulfoxide (DMSO) at 21.7 mg/mL and diluted in serum-free medium to a final concentration of 40% DMSO). Vehicle and FH535 were administered by intraperitoneal injection every other day. Tumors were measured using an optical caliper and tumor size was calculated using the formula: 0.5 × length × (width)^2^. Mice were euthanized at the end of the experiment or when reaching humane end-point following AVMA guidelines. Humane end-points included animals with tumors exceeding 20 mm in maximum diameter, with ulcerated tumors, more than 20% body weight loss, impaired mobility, labored breathing or with a body condition score below 2 [27].

### Hematoxylin and Eosin (H&E) and immunohistochemistry of explanted tumors

Tumors from the xenograft model were formaldehyde fixed and paraffin-embedded and were used to performed H&E staining and immunohistochemistry of Ki-67 according to standard procedures.

### Western blot analyses

Cell lysates were prepared in ice-cold RIPA buffer with freshly added protease inhibitor cocktail (ThermoFisher, USA). Protein concentration was determined using the BCA Protein Assay (ThermoFisher, USA). Cellular proteins (20–40 μg) were separated on SDS-polyacrylamide gel and transferred to PVDF membrane (ThermoFisher, USA). Primary antibodies are described in S1 Table. All primary antibodies were used at 1:1000 dilution dilution with exception of the β-actin antibody at 1:10000 following manufacturer recommendations. Proteins were detected by incubating with horseradish peroxidase-conjugated antibodies (Cell Signaling Technology, USA). Specific bands were visualized with enhanced chemiluminescence reagent (BioRad, USA) and quantified using the ImageJ software (Bethesda, Maryland, USA).

### Quantitative real-time RT-PCR

Total RNA was extracted with miRNeasy mini kit (Qiagen, Germany), and the corresponding cDNA was produced using iScript cDNA synthesis kit (BioRad, USA) from 1 μg of total RNA. Real time quantitative PCR (RT-qPCR) was performed using SsoAdvanced Universal SYBR Green supermix (BioRad, USA) with specific primers: p62 (SQSTM1: 5’-AAGCCGGGTGGGAATGTTG-3’ and 5’-GCTTGGCCCTTCGGATTCT-3’); c-MYC (cMYC: 5’-TTTTCGGGTAGTGGAAAACCAGC-3’ and 5’-AGTAGAAATACGGCTGCACCGA-3’), Survivin (BIRC5, 5’-CAAGGAGCTGGAAGGCTGG-3’ and 5’-GTTCTTGGCTCTTTCTCTGTCC-3’), AXIN 2 (AXIN2: 5’-CAGCAGAGGGACAGGAATCATT-3’ and 5’-GCCAGTTTCTTTGGCTCTTTGTG-3’), Cyclin D1 (5’-CCND1: GGATGCTGGAGGTCTGCGA-3’ and 5’-TAGAGGCCACGAACATGCAAGT-3’), and b2-microglobulin (B2M: 5’-GACTTTGTCACAGCCCAAGATAG-3’ and 5’-TCCAATCCAAATGCGGCATCTTC-3’). Transcript levels were normalized to β-actin or β-2-macroglobulin level as indicated using the ΔΔCt method.

### Metabolic analysis

Oxygen Consumption Rates (OCR) were measured on an XF-96 Extracellular Flux Analyzer (Seahorse Bioscience) using the protocol and conditions optimized for HCC cells as previously described by our group [28]. Briefly, the experiments were performed by seeding 10,000 and 20,000 Huh7 cells per well in XFe 96 well-plates 36 h before the experiment. Cells were treated for 24 h with FH535, FH535-N or vehicle control. In OCR experiments the media was supplemented with 25 mM Glucose and 1mM Pyruvate just before the assay. After minimal incubation time (~20–30 min in non-CO_2_ 37°C incubator) mitochondrial stress test was initiated. During the assay, 4 different drugs with the following final concentrations were injected to all of the 96 wells: 1) Port A—1 μM Oligomycin A, 2) Port B—1.5 μM Carbonyl cyanide 4-(trifluoromethoxy) phenylhydrazone (FCCP), 3) Port C—200 mM Etomoxir, and 4) Port D—mixture of 1 μM Rotenone and 1 μM Antimycin A. All reagents used in the Seahorse experiments were purchased from Sigma-Aldrich. Analyses of data were performed with Wave 2.2 software (Seahorse Bioscience), Excel (Microsoft Office 2013) and Prism 7.0 (GraphPad Software).

### Autophagy assay

Autophagy responses were monitored with the Cyto-ID autophagy detection reagent 2.0 (Enzo Life Sciences, USA) according to the manufacturer’s instructions. Huh7 and PLC/PRF/5 cells were seeded in 12-well plates and treated the following day with drugs or DMSO vehicle control at the indicated concentrations. CQ was added to the corresponding wells to a final concentration of 50 μM 8 h prior harvesting. After 40 h of drug treatment, cells were collected and assessed for viability with Zombie Violet dye solution (BioLegend, USA) followed by Cyto-ID autophagy detection reagent staining. Flow cytometry analyses were performed in a BD LSRII at the Flow Cytometry and Cell Sorting Shared Resource Facility of the University of Kentucky Markey Cancer Center and data were analyzed with the FlowJo Software version 7.6.5 (Tree Star, USA). The autophagic flux was quantified by subtracting the Cyto-ID MFI value of the sample without CQ from the Cyto-ID MFI value of the sample with CQ for each condition according to the formula:

ΔLC3II=LC3II(+CQ)−LC3II(−CQ) (1)

Analysis of Cyto-ID MFI was performed on live cells (Zombie negative stained population).

### β-Catenin Knockdown

Huh7 cells were transfected using lipofectamine RNAiMAX reagent (Invitrogen, USA) with Silencer Select Negative Control No. 1 siRNA (Ambion, USA) or β-catenin siRNA (s438, Ambion, USA) at a final concentration of 10 nM.

### 2,5-dichloro-*N*-(4-nitronaphthalen-1-yl)benzenesulfonamide (FH535-N)

**Fig 1:**
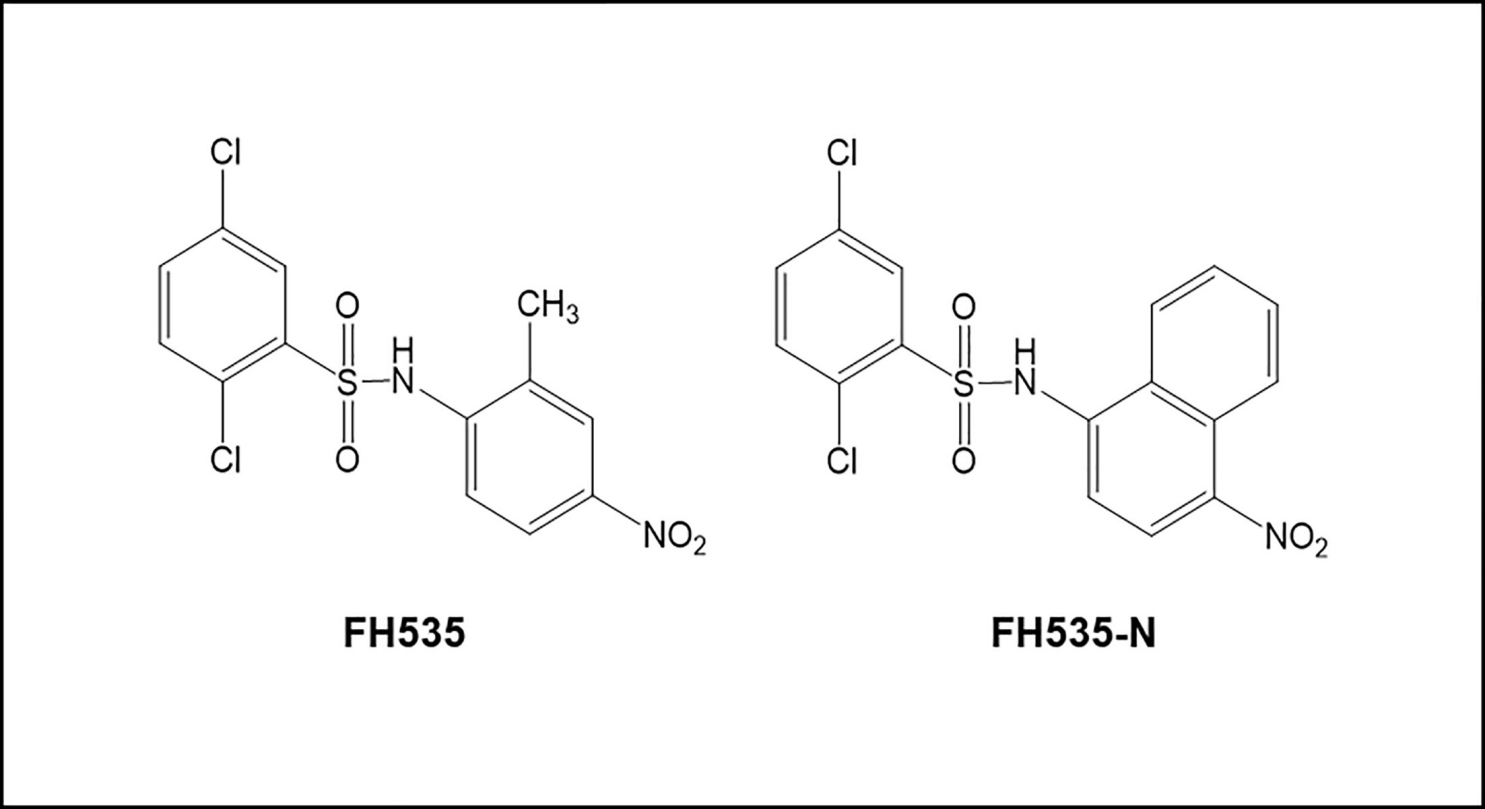
Structure of FH535 (2,5-dichloro-N-(2-methyl-4-nitrophenyl)benzenesulfonamide) and FH535-N (2,5-dichloro-N-(4-nitronaphthalen-1-yl)benzenesulfonamide).

The synthesis of 2,5-dichloro-*N*-(4-nitronaphthalen-1-yl)benzenesulfonamide was described previously by Kril, *et al*., in which the *N*-(2-methyl-4-nitrophenyl) group of FH535 was replaced with a *N*-(4-nitronaphthyl) group (Fig 1)[29].

### [^3^H]-thymidine incorporation assay

Huh7, PLC/PRF/5 and Hep3B cells were plated in 96-well plates at 3000–4000 cells/well, treated with the concentrations indicated of FH535 or FH535-N, as single agents or in combination with sorafenib, and cultured for 72 h. ^3^H-thymidine incorporation assay was performed as described previously [24, 30].

### Apoptosis assay

Apoptosis assay was performed in Huh7 and PLC/PRF/5 cells treated 48 h with DMSO vehicle control or the indicated doses of FH535, FH535-N alone or in combination with sorafenib. Cells were harvested and stained with the APC Annexin V apoptosis detection kit with PI (BioLegend, USA) according to the manufacturing instructions followed by flow cytometry analysis. Flow cytometry data was acquired with an LSRII instrument (BD-Biosciences) and analyzed with FlowJo software (Tree Star).

### Dual luciferase assay

Cells were plated at 1x10^5^ cells/well in 24-well plates and transiently transfected with 490 ng of luciferase-reporter plasmid and 10 ng of phRL-TK per well using the Turbofect transfection reagent (Thermo Scientific, USA). After 5–6 h post-transfection, cells were treated with drug(s) or DMSO vehicle control for 36 h in the presence or absence of LiCl (10 mM). Luciferase assays were performed using the Dual Luciferase Assay System (Promega, USA) according to manufacturer’s instructions.

### Statistical analysis

Data was reported as mean ± SD of triplicate experiments (except where indicated). Statistical analyses were performed using GraphPad PRISM 7.0. Statistical significance of differences between two groups was analyzed using Student’s *t*-test or ANOVA with post-hoc Tukey HSD accordingly. In all analyses, *p*<0.05 was considered a statistically significant difference.

## Results

### FH535 inhibits the growth of xenograft tumors in mice

**Fig 2:**
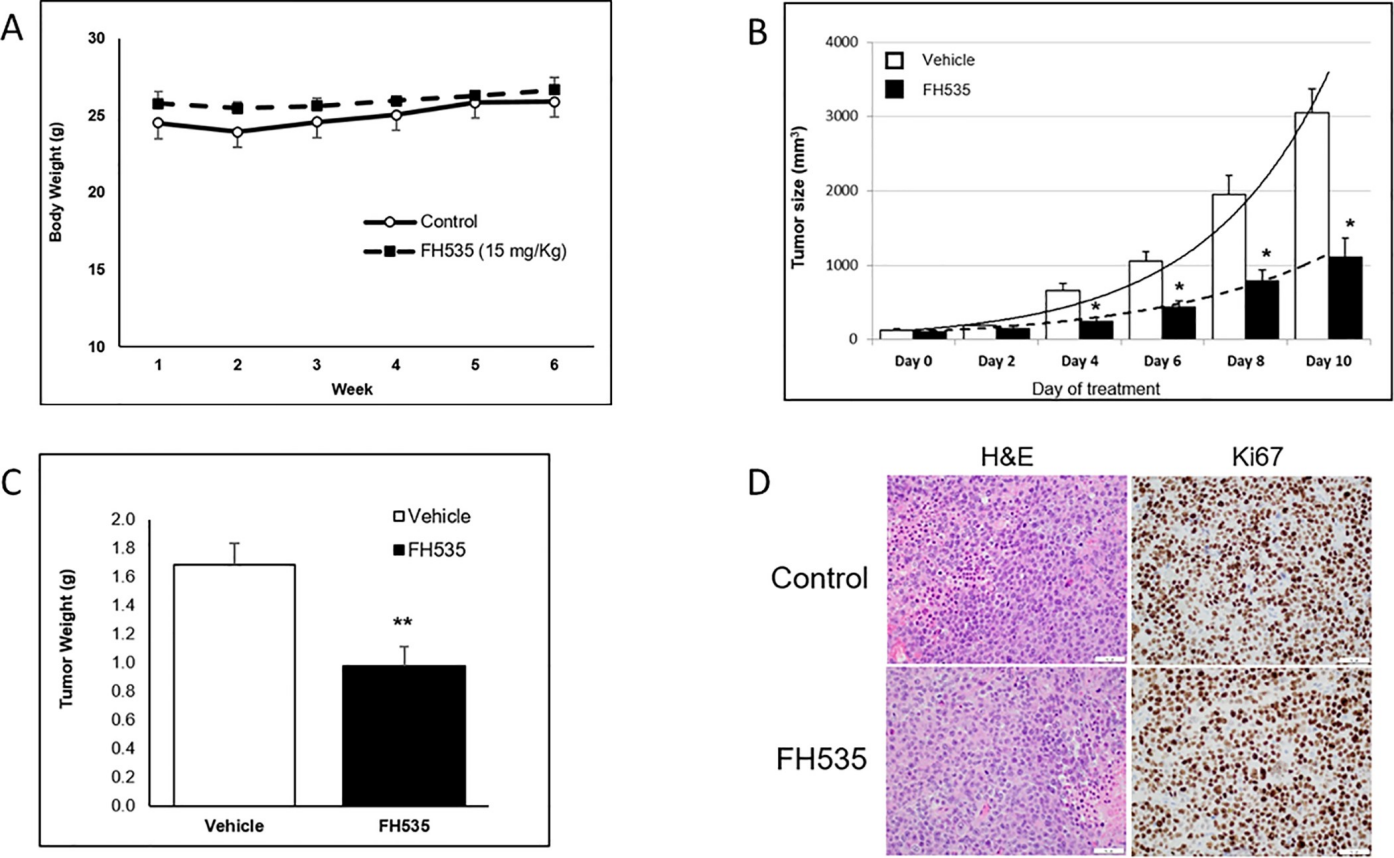
FH535 effect *in vivo*. (A) Mice body weight after FH535 treatment. C57BL/6 mice (n = 5) were treated by intraperitoneal injection with 15 mg/Kg of FH535 or DMSO vehicle control every 4 days for 6 weeks. Mice were monitored before injections for signs of body weight loss, impaired mobility, labored breathing and body score based on the Ullman-Culleré MH, Foltz CJ method [27]. (B-D) FH535 reduces tumor growth *in vivo* in a xenograft tumor model. Huh7 cell were injected subcutaneously on the right flank of athymic nude mice. FH535 (15 mg/Kg) or vehicle (DMSO) were administrated by intraperitoneal injection every other day when tumor size reached 100 mm^3^. (B) Tumor growth was monitored every other day until day 10 of starting treatments when mice were euthanized according to the AVMA guidelines, *p< 0.05 (n = 5, each group); (C) Tumor weight of excised tumors after 10 day treatment with FH535 reduced the tumor weight in 42 ± 8% compared to vehicle treatment, **p< 0.001 (n = 4, each group). (D) H&E and ki67 staining from one representative tumor of each group treatment. Pictures were taken at 400X magnification. H&E staining showed poorly differentiated carcinoma comprised of sheets of epithelioid cells with increased N/C ratio, enlarged nuclei with prominent nucleoli, high mitotic activity and tumor necrosis (lower right corner of the picture for FH535, and left upper corner and left mid area of the picture for control group). The Ki-67 immunohistochemical staining highlights very high mitotic index with nuclear staining in more than 95% of the viable neoplastic cells for both groups.

We previously showed that FH535 decreased the proliferation of different, human HCC cell lines, including Huh7 cells [24]. To validate further the *in vivo* anti-tumor effect of FH535, we performed a gross-toxicity assay in mice with FH535 doses ranging from 0 to 30 mg/kg. We first demonstrated that intraperitoneal injections up to 15 mg/kg of FH535 for a period of 5–6 weeks did not induce major signs of body distress or toxicity such as weight loss, decreased ambulatory ability, labored respiration or dehydration (Fig 2A). Next, we evaluated the *in vivo* anti-tumor activity of FH535 in a Huh7 tumor xenograft model. When HCC tumors reached a volume of 100 mm^3^, mice were injected with DMSO vehicle (control group) or 15 mg/kg of FH535 every other day. After only four days of treatment, the tumor volumes of FH535-treated mice were already significantly reduced compared to control group (p<0.05) (Fig 2B and 2C). This result demonstrated the efficacy of the FH535 *in vivo* on the progression of HCC tumor growth. We also performed Hematoxylin and Eosin staining to assess tumor characteristics and showed that tumors in both groups were poorly differentiated HCC. We evaluated proliferation index using immunohistochemistry with Ki-67 expression, which demonstrated a proliferation index greater than 95% in both groups (Fig 2D).

### FH535 affects autophagy in HCC cells

**Fig 3:**
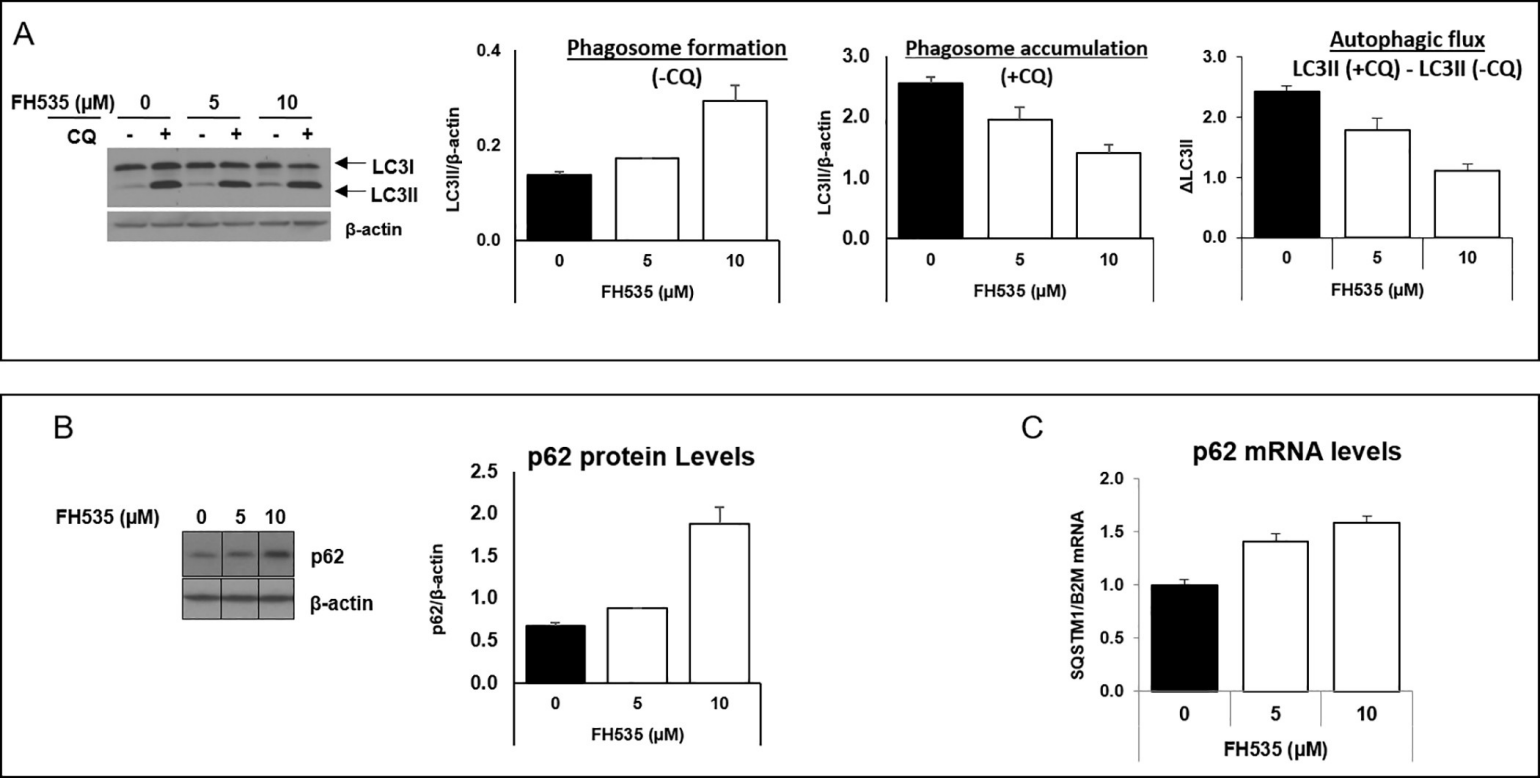
FH535 regulates autophagic activity in HCC cells. Western blot analysis of Huh7 cell after 40 h treatment with FH535 at indicated concentrations in absence (-CQ) or presence (+CQ) of 50 μM chloroquine for 8h. LC3B (A) and p62 (B) were used as autophagy markers for western blot analysis. Band intensity were estimated using ImageJ software. Autophagic flux was determined by subtracting the band intensity of LC3B II western blot in presence of CQ and the corresponding treatment in absence of CQ which is referred as ΔLC3II (LC3II (+CQ)—LC3II (-CQ)) (A, right panel). mRNA p62 expression levels were assessed by RT-qPCR in absence of CQ (C).

Increasing evidence for the crosstalk between Wnt/β-catenin and autophagy prompted an evaluation of the linkage between the anti-proliferative effect of FH535 on HCC and autophagy modulation. To investigate this possibility, we first examined LC3 expression levels as a marker of autophagic activity (Fig 3A). Treatment of Huh7 cells with FH535 increased the lipid-bound expression of LC3II levels in a dose-dependent manner in comparison with control-treated cells, a finding that indicated an accumulation of autophagosomes (Fig 3A, left panel). Consistent with the results observed for LC3II, western blot analyses indicated that p62, another autophagy marker, was also increased in FH535-treated cells (Fig 3B). In addition, the knockdown of β-catenin in Huh7 cells increased the expression of both LC3II and p62, which is consistent with the targeting of β-catenin as potential mechanism of action of FH535 (S1 Fig). In this context, β-catenin was reported as a transcriptional repressor of p62 [31], and as expected, our results verified the increase of p62 mRNA levels in the presence of FH535 (Fig 3B and 3C).

### FH535 modulates autophagy flux in HCC cells

**Fig 4:**
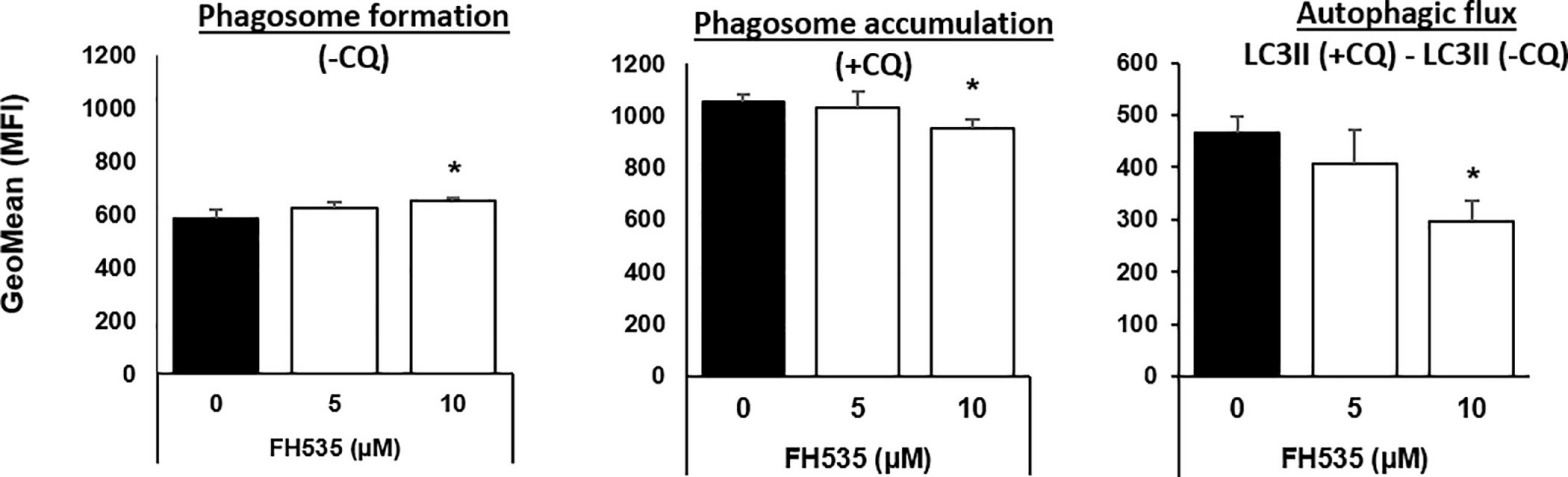
FH535 regulates autophagic flux in Huh7 cells. Autophagic activity of Huh7 cells after 40 h FH535 treatment in absence (-CQ) or presence (+CQ) of 50 μM CQ (8h) was determined by flow cytometry analysis using the Cyto-ID autophagy detection reagent. Results are shown as GeoMean ± SD from viables cells (Zombie negative population). Autophagic flux was determined by the difference in GeoMean between cells treated with CQ and corresponding treatment in absence of CQ also referred as ΔGeoMean (GeoMean (+CQ)—GeoMean (-CQ)) (right panel). *: p ≤ 0.05.

The accumulation of LC3II and p62 observed in FH535-treated cells were consistent with an effect on autophagy. This effect was attributed to changes in either autophagosome formation, the autophagic flux, or both. To discriminate among these processes, we analyzed the induced accumulation of LC3II protein levels after blocking the autophagosome degradation with CQ. As expected, the addition of CQ enhanced LC3II accumulation in both control and drug-treated cells compared to the corresponding experiments without CQ (Fig 3A, middle panel). However, the progressive increase of LC3II levels in FH535-treated cells alone (Fig 3A, first and second panels) and the reduced accumulation of LC3II in the presence of CQ (first, third and fourth panels) reflected the alteration of the autophagic activity in FH535-treated cells. To further support these findings, the Cyto-ID autophagy detection assay revealed a reduced accumulation of autophagic vesicles in response to FH535 treatment after addition of CQ (Fig 4). Together, these results are consistent with the reduced autophagic flux in FH535-treated cells.

### FH535-N on HCC proliferation, apoptosis and β-catenin pathway

**Fig 5:**
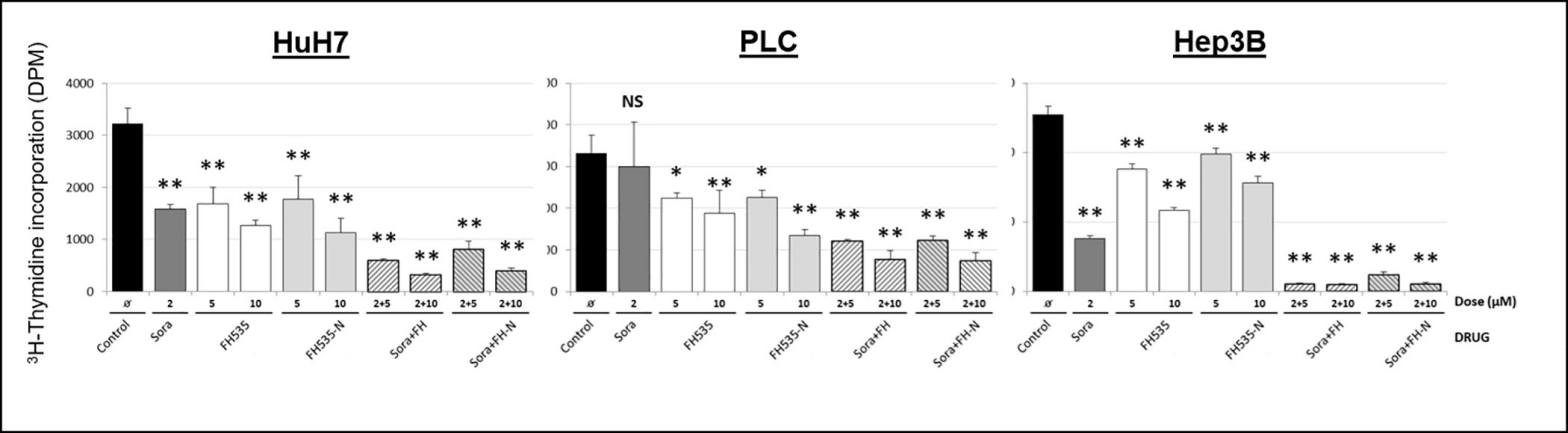
Effect of FH535-N on HCC cells proliferation. Cell proliferation was measured on Huh7, PLC/PRF/5 and Hep3B cells using 3H-thymidine incorporation after 72 h treatment with FH535 or FH535-N alone or in combination with sorafenib at the concentrations indicated. Results are represented as mean ± SD, n = 4. *: p ≤ 0.05, **p ≤ 0.001.

**Fig 6:**
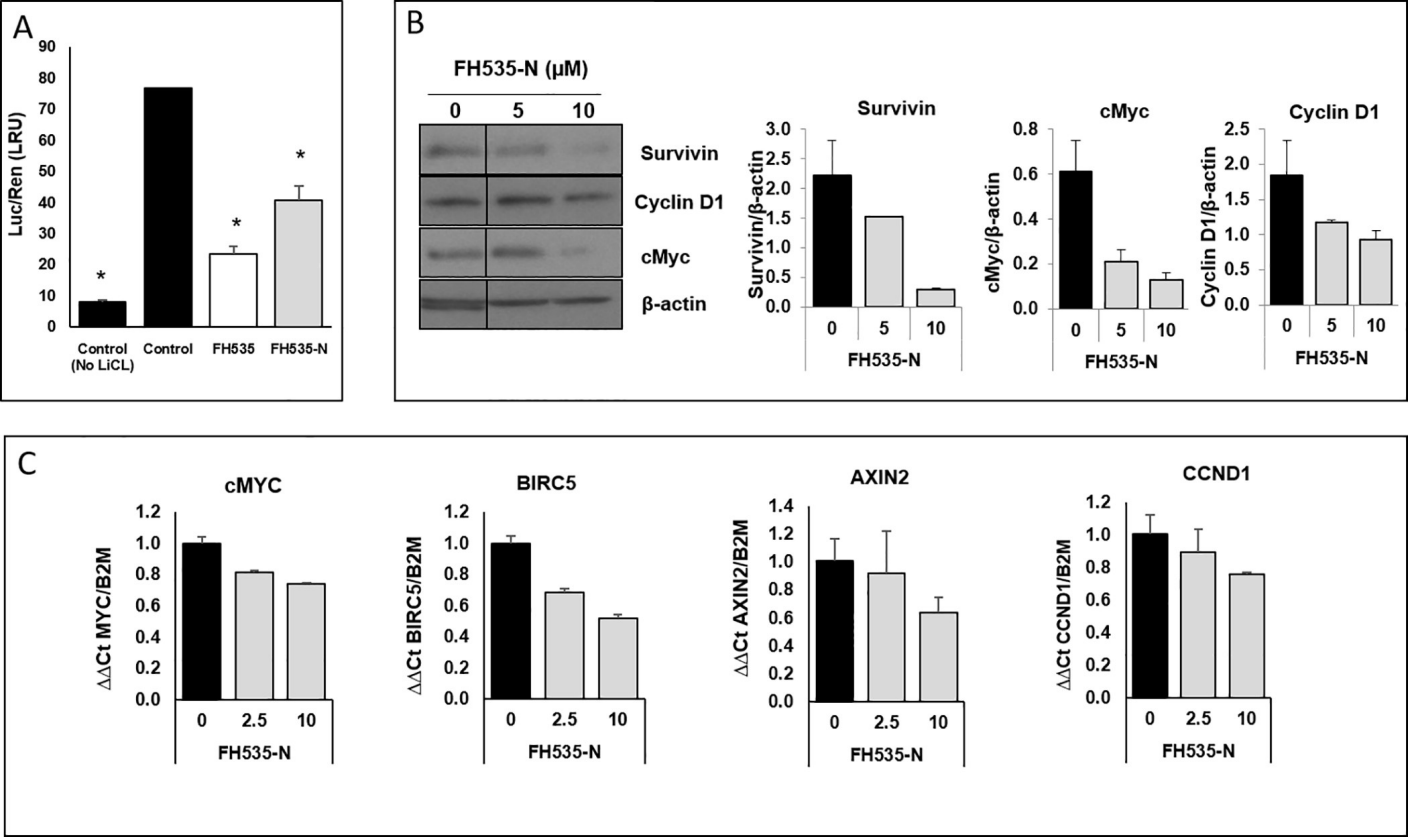
Effect FH535-N on inhibition of Wnt/β-catenin pathway. (A) Effect of FH535-N on TOPFlash activation. Huh7 cells were co-transfected with Top-Flash and phRL-TK plasmid. After 5 h of transfection, cells were treated with vehicle, FH535 or FH535-N at the concentration indicated in presence of 10 mM LiCl. Vehicle in absence of LiCl was used as control for basal levels of Wnt/β-catenin activity. Results are represented as mean ± SD, n = 3. #: p ≤ 0.05, *: p ≤ 0.001. (B-C). Effect of FH535-N on expression of β-catenin targets. Protein expression levels of downstream β-catenin targets from Huh7 cells treated with FH535-N for 36 h were determined by western blot analysis (B, left pannel). Densitometry analysis was performed using ImageJ software (B, right pannel). mRNA expression of downstream β-catenin targets from Huh7 cells treated with FH535-N for 36 h were determined by RT-qPCR (D).

**Fig 7:**
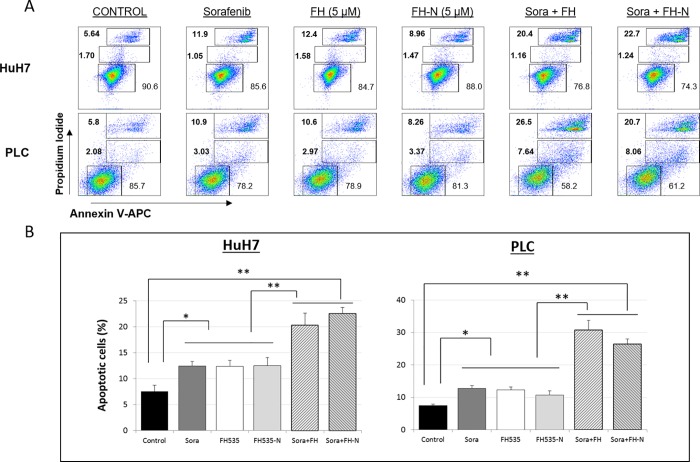
Effect of FH535-N alone or in combination with sorafenib on apoptosis of HCC cells. Analysis of apoptosis by Annexin V-APC/propidium iodide (PI) double staining of HuH7 and PLC/PRF/5 cells after 48 h treatment at the concentration of FH535, FH535-N and sorafenib indicated. (A) Two-color flow cytometry dot plots show the percentages of living cells as negative for both annexin V and PI; early-stage apoptotic cells as the populations testing Annexin V positive and PI negative, and late-stage apoptotic/necrotic cells as double-positive cells. Results are represented in (B) as mean ± SD, n = 3. *: p ≤ 0.05, **: p ≤ 0.001.

**Fig 8:**
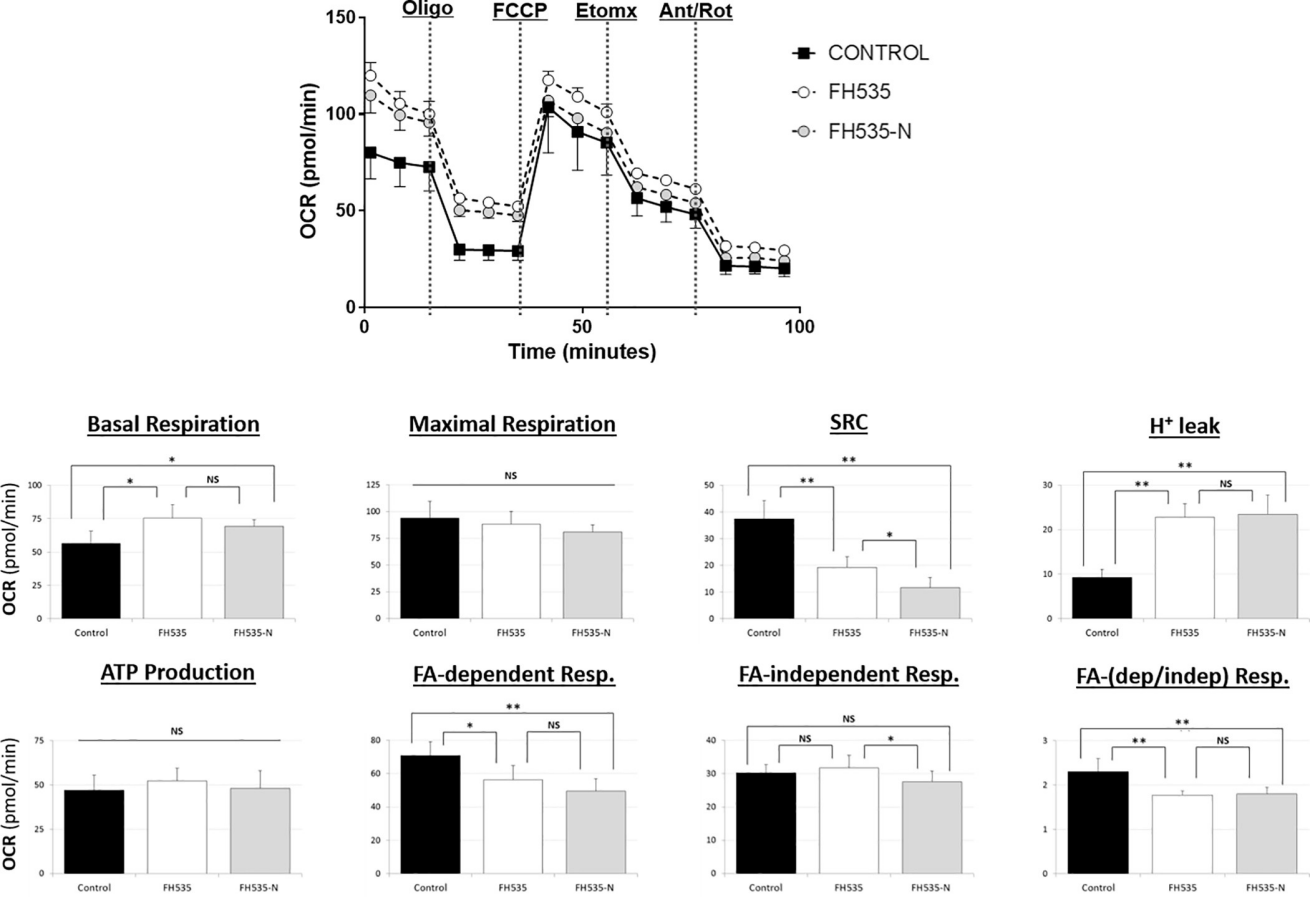
Mitochondrial respiration changes induced after 24 h-treatment of Huh7 cells with FH535, FH535-N alone or in combination with sorafenib. Representative OCR profiles of Huh7 cells shown as percentage change with respect to the OCR levels after addition of the ATP-synthase inhibitor Oligomycin (O). The parameters of ATP turnover, proton leak, and spare respiratory capacity were calculated as area under the curve (AUC) values as described in Materials and Methods section. Data are shown as mean ± SEM, n = 6–8.*: p < 0.05 and **: p<0.001. Statistical comparisons were performed using one-way ANOVA and Dunnett’s multiple comparisons test and pairwise comparisons with Student’s t test. (NS = non-significant; p≥0.05).

**Fig 9:**
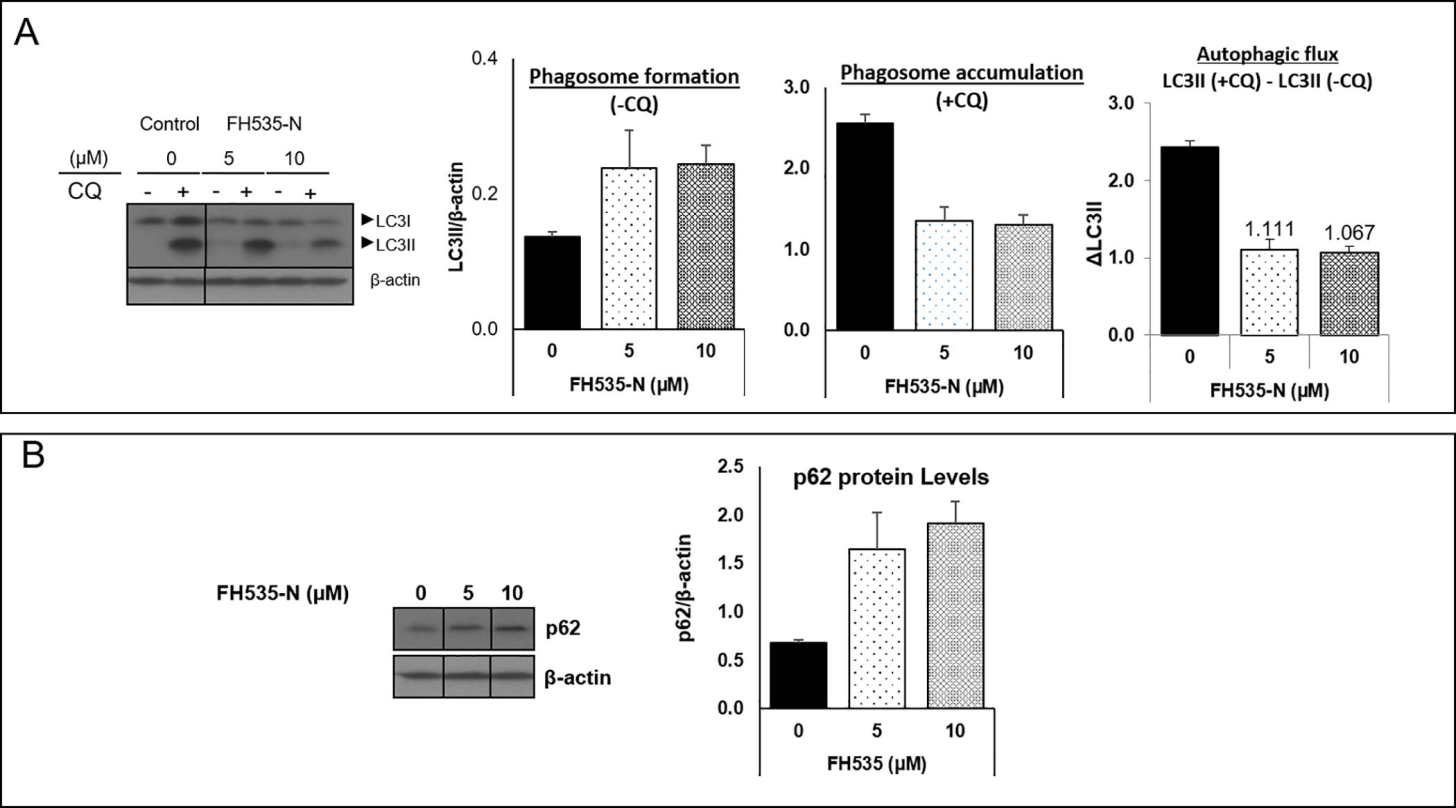
FH535-N regulates autophagic activity in HCC cells. Western blot analysis of Huh7 cell after 40 h treatment with FH535-N at indicated concentrations in absence (-CQ) or presence (+CQ) of 50 μM chloroquine for 8h. (A) Protein expression levels of LC3BII. Autophagic flux was determined by subtracting the band intensity of LC3B II western blot in presence of CQ and the corresponding treatment in absence of CQ which is referred as ΔLC3II (LC3II (+CQ)—LC3II (-CQ)). (B) Protein expression levels of p62 in absence of CQ. Band intensity from Western blots were estimated using ImageJ software.

**Fig 10:**
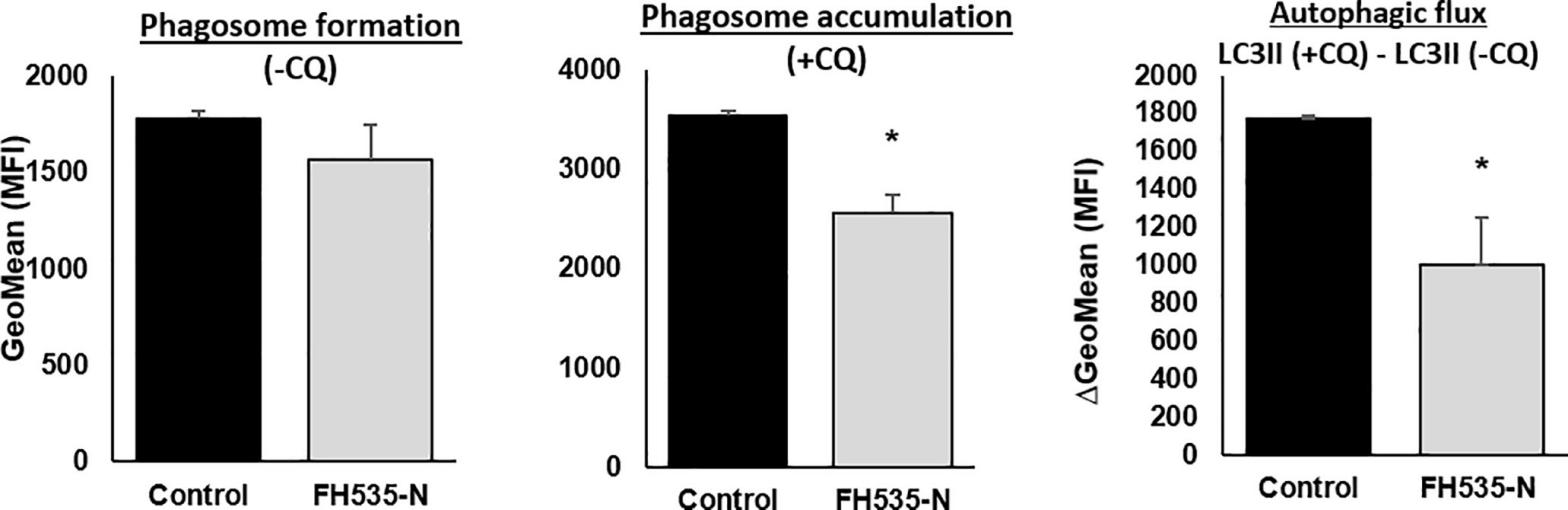
FH535-N regulates autophagic flux in Huh7 cells. Autophagic activity of Huh7 cells after 40 h FH535 treatment in absence (-CQ) or presence (+CQ) of 50 μM CQ (8h) was determined by flow cytometry analysis using the Cyto-ID autophagy detection reagent. Results are shown as GeoMean ± SD from viables cells (Zombie negative population). Autophagic flux was determined by the difference in GeoMean between cells treated with CQ and corresponding treatment in absence of CQ also referred as ΔGeoMean (GeoMean (+CQ)—GeoMean (-CQ)) (right panel). *: p ≤ 0.05.

**Fig 11:**
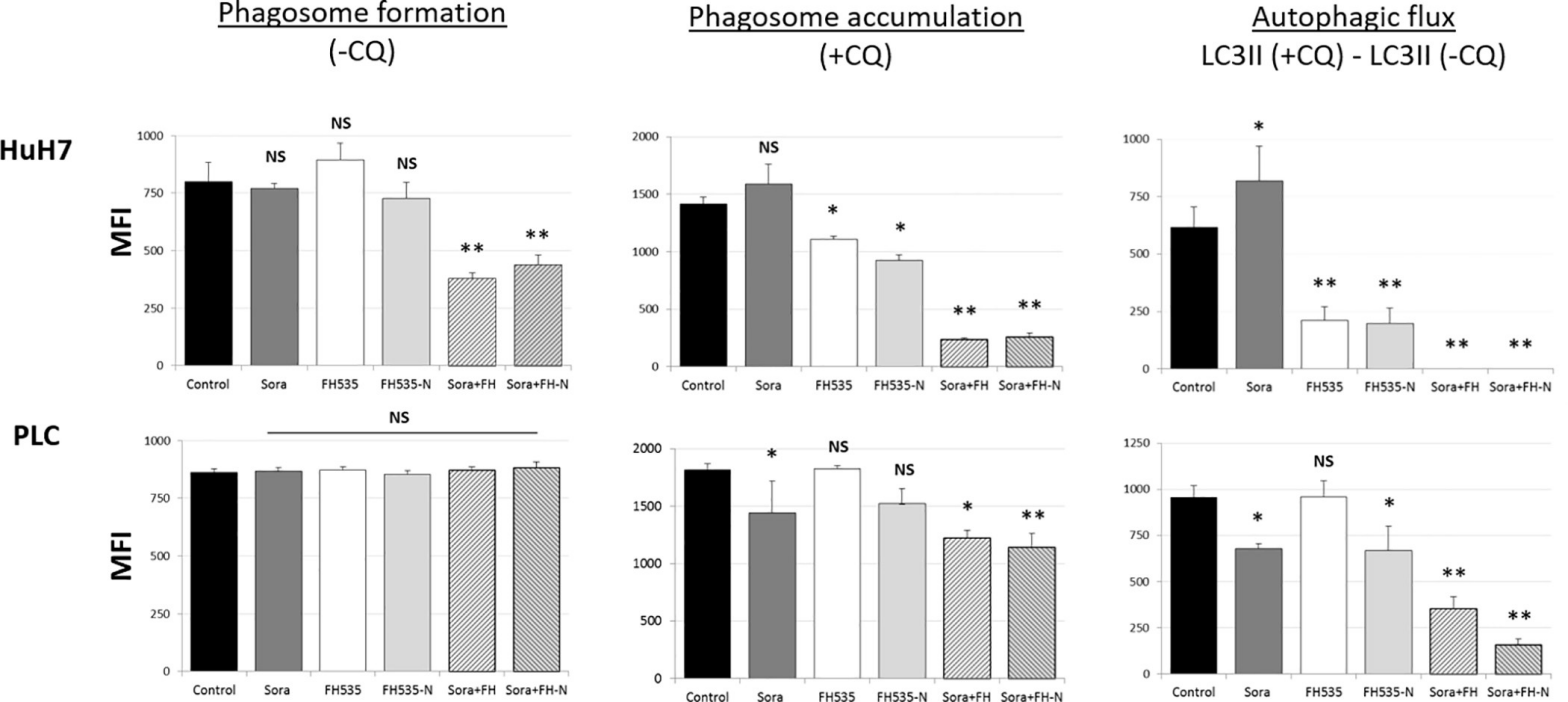
FH535-N regulates autophagic flux in Huh7 and PLC/PRF/5 cells. Autophagic activity of Huh7 cells after 40 h FH535 treatment in absence (-CQ) or presence (+CQ) of 50 μM CQ (8h) was determined by flow cytometry analysis using the Cyto-ID autophagy detection reagent. Results are shown as GeoMean ± SD from viables cells (Zombie negative population). Autophagic flux was determined by the difference in GeoMean between cells treated with CQ and corresponding treatment in absence of CQ also referred as ΔGeoMean (GeoMean (+CQ)—GeoMean (-CQ)) (right panel).*: p < 0.05 and **: p<0.001. (NS = non-significant; p≥0.05).

We previously described the synthesis of FH535 derivatives from commercially available halogen-substituted arylsulfonyl chlorides and aryl amines [29]. 2,5-Dichloro-N-(4-nitronaphthalen-1-yl)benzenesulfonamide (FH535-N) inhibited cell proliferation of Huh7, PLC and Hep3B cells (Fig 5) and reduced the Wnt/β-catenin transcriptional activity as demonstrated by using a TOP-Flash TCF4-dependent luciferase reporter assay (Fig 6A) as well as the expression of known downstream Wnt/β-catenin targets genes (Fig 6B and 6C). FH535-N demonstrated significant increased rate of apoptosis in Huh7 and PLC/PRF/5 (Fig 7). In a previous article our group demonstrated the targeting of FH535 on mitochondrial respiration activity. Based on these results we performed a comparative study of the effects of FH535 and the derivative FH535-N on OCR. Our results, showed that both drugs induced similar inhibition of Spare Respiratory Capacity (SRC) and enhanced Proton Leak. These findings indicate similar alteration of the metabolic plasticity and increased oxidative stress of HCC cells treated with FH535 or FH535-N (Fig 8). We analyzed the expression levels of LC3II and p62 in Huh7 cells after treatment with FH535-N in the presence and absence of CQ. Similar to the results observed with FH535, FH535-N also increased LC3II and p62 protein levels (Fig 9A and 9B). Addition of CQ demonstrated accumulation of LC3II with FH535-N and CQ treatment, as expected. However, this accumulation in the combination treatment is reduced at higher doses of FH535-N consistent with the results of FH535-treated cells. (Fig 9A right panel). These results were consistent with a reduction in the autophagic flux in response to FH535-N in a fashion that mirrored the effects described for FH535. In addition, this possibility was supported by the results of FH535-N in Cyto-ID autophagy readouts (Fig 10). Similar results were observed in PLC/PRF/5 cells (Fig 11 and S2 Fig). Overall, our data demonstrated that the anti-proliferative effects of FH535 and its derivative, FH535-N, on HCC cells are associated with the regulation of autophagic processes.

### FH535 and FH535–N in combination with sorafenib on HCC cell proliferation, apoptosis and autophagy

Our group have previously demonstrated a synergistic effect on cell proliferation using FH535 in combination with sorafenib. Due to these findings, we assessed the effect of drug combination of FH535 or FH535-N with sorafenib on HCC cell proliferation, apoptosis and autophagic flux. We found that FH535 and FH535-N have an additive effect on HCC cell proliferation of Huh7, PLC/PRF/5 and Hep3B in combination with sorafenib (Fig 5). We have also observed a significant increase in apoptosis measured by Annexin V/PI using the combination treatments (FH535/sorafenib, FH535-N/sorafenib). This effect of the drug combination was more pronounced that the one seen with FH535, FH535-N or sorafenib alone (Fig 7). FH535/sorafenib and FH535-N/sorafenib drug combination produced a significant decreased in the authophagic flux measured by CytoID (Fig 10). We also used HCC cell lines Huh7 and PLC to perform WB assay to assess changes in P62 and LC3 expression with monotherapy and combination treatment. We found an accumulation of P62 and LC3-II in the presence of drug combination (FH535/sorafenib and FH535-N/sorafenib) (S2 Fig).

## Discussion

Aberrant activation of the Wnt/β-catenin pathway occurs in numerous malignancies, including HCC [6, 11–14]. The poor prognosis and disease progression in liver cancer typically involves the upregulation of the Wnt/β-catenin pathway [7], and recent efforts focus on the development of new compounds targeting this and other signaling pathways as effective therapeutic alternatives for advanced HCC. The *N*-aryl benezenesulfonamides, such as FH535, inhibits the Wnt/β-catenin signaling pathway and the PPARs δ and ɣ with demonstrated anti-proliferative effect against pancreatic cancer, breast cancer, colorectal carcinoma and HCC cells [21, 24, 32, 33]. FH535 also sensitizes and reverses the epithelial-mesenchymal transition phenotype of radio-resistant esophageal cancer cells [34]. *In vivo*, FH535 effectively suppresses growth and angiogenesis in pancreatic cancer and decreases tumor burden and progression in colorectal cancer [21, 33]. We now demonstrate the potent effects of FH535 on HCC tumor progression *in vivo* using a mouse xenograft model while showing no significant drug toxicity in the host.

Although, there is important pre-clinical evidence for the anti-cancer effects of FH535, the mechanism of action of this drug remains poorly understood. We recently demonstrated that FH535 induces changes in mitochondrial membrane potential and overall mitochondrial health in HCC tumor cells [23]. FH535 targets specifically the electron transport chain complexes I and II and results in defective mitochondrial respiration [23]. Since mitochondrial dysfunction and Wnt/β-catenin signaling affect the regulation of the autophagy process [35], this study reports on the anti-tumor effect of FH535 and its derivative FH535-N on HCC cells through the modulation of the autophagic activity.

Compared to untreated-control cells, our results demonstrate that FH535 increased LC3II and p62 levels in HCC cells, a finding that is indicative of autophagosomal accumulation by the increase in autophagosome formation and/or by a defective lysosomal degradative machinery. A modest CQ-induced increase in autophagosome accumulation occurs in FH535-treated cells, together with the reduced ΔLC3II levels in western blots, an additional finding that is consistent with an impaired autophagic flux. This may contribute to the accumulation of dysfunctional mitochondria and account for the increased apoptosis in FH535-treated cells. Future studies will assess the involvement of FH535 on autophagosomal formation on HCC cells as suggested by the enhancement of *p62/SQSTM1* gene expression.

Several studies reveal the complex interplay between Wnt/β-catenin signaling and autophagy [31, 36–39]. The coordinated regulation of Wnt/β-catenin signaling and autophagy processes occurs in different types of cancers, including the cytotoxic effect of resveratrol on breast cancer cells [40] and the reduced gemcitabine-induced apoptosis in human osteosarcoma cells [41]. In this regard, the inhibition of Wnt/β-catenin pathway induces the accumulation of autophagic proteins such as LC3-II, ATG7, Beclin-1 and p62 proteins [38, 42]. Reciprocally, induction of autophagy regulates the Wnt/β-catenin pathway by targeting the clearance of β-catenin and other proteins involved in Wnt signaling such as the Dishevelled protein [31, 43]. In agreement with these studies, our results show that FH535 treatment induces the accumulation of LC3II and p62 proteins as well as *p62/SQSTM1* mRNA and suggests that the effect of FH535 on autophagy links to the inhibition of Wnt/β-catenin signaling. In support of this possibility, the β-catenin knockdown in HCC cells also exhibits a subsequent increase in LC3II and p62 protein levels.

*N*-Aryl benezenesulfonamides, such FH535 and FH535-N exhibit significant anti-cancer effects [29]. In this study, FH535 and FH535-N produce similar anti-proliferative activity in HCC cells. Furthermore, both compounds target the Wnt/β-catenin signaling pathway as indicated by β-catenin-dependent reporter assays (Fig 6A) as well as the reduced expression of endogenous downstream β-catenin target genes (Fig 6B and 6C). Additionally, FH535-N induces the accumulation of autophagic proteins p62 and LC3II and impairs the autophagic flux in Huh7 cells. These results provide further evidence that FH535-based derivatives warrant additional development as anti-cancer drug candidates for HCC treatment. Moreover, the synergistic effects of FH535 and sorafenib on the inhibition of HCC cell proliferation and survival was associated to the distinct targeting of both drugs on the mitochondrial function and metabolic pathways [23]. Inhibition of autophagy by ATG7 knockdown or CQ treatment sensitizes HCC cells to sorafenib by enhancing apoptosis [18]. Likewise, and consistent with these reports, our findings indicate that the inhibition of autophagic flux by FH535 and FH535-N contributes, at least partially, to the synergistic effects observed using FH535 in combination with sorafenib. We also demonstrate an additive effect of drug combination therapy using FH535 or FH535-N with sorafenib on HCC cell proliferation and apoptosis. Importantly, our findings revealed a significant increase in autophagic disruption caused by these combinatory treatments compared to FH535, FH535-N or sorafenib alone.

In conclusion, our data demonstrate potent anti-tumor effects of FH535 *in vivo* at dosage levels (15 mg/kg/day) that produce no gross toxicity in the mice. These studies also reveal a contributing mechanism for the anti-tumor action of FH535 with the Wnt/β-catenin-mediated regulation of the autophagy process. Further studies are warranted to assess the efficacy of FH535 and its derivatives either alone or in combination with conventional therapies as rational therapeutic alternatives for HCC treatment.

## Supporting information

S1 Table Antibodies used for western blot. (TIF)

S1 Fig β-catenin knockdown induced changes in LC3II and p62 protein levels in Huh7 cells. Western blot analysis of LC3BII and p62 protein levels of Huh7 transiently transfected with a β-catenin (β-cat) or control (Ctrl) siRNA.
(TIF)

S2 Fig Effect of FH535 and FH535-N in combination with sorafenib on the protein levels of autophagy markers LC3B II and p62. (TIF)

## Abbreviations

HCC: Hepatocellular carcinoma
CQ: chloroquine
FH535: 2,5-dichloro-N-(2-methyl-4-nitrophenyl)benzenesulfonamide
FH535-N: 2,5-dichloro-N-(4-nitronaphthalen-1-yl)benzenesulfonamide
