# Supplementary figures and images for: Autophagic flux modulation by Wnt/β-catenin pathway inhibition in hepatocellular carcinoma
Source: PLoS One. 2019 Feb 22;14(2):e0212538. doi: 10.1371/journal.pone.0212538 (PMC6386480; doi:10.1371/journal.pone.0212538)

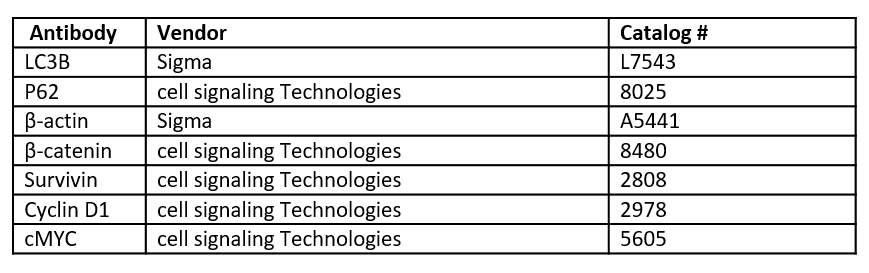

Supplement: S1 Table — (TIF) [file pone.0212538.s001.tif]

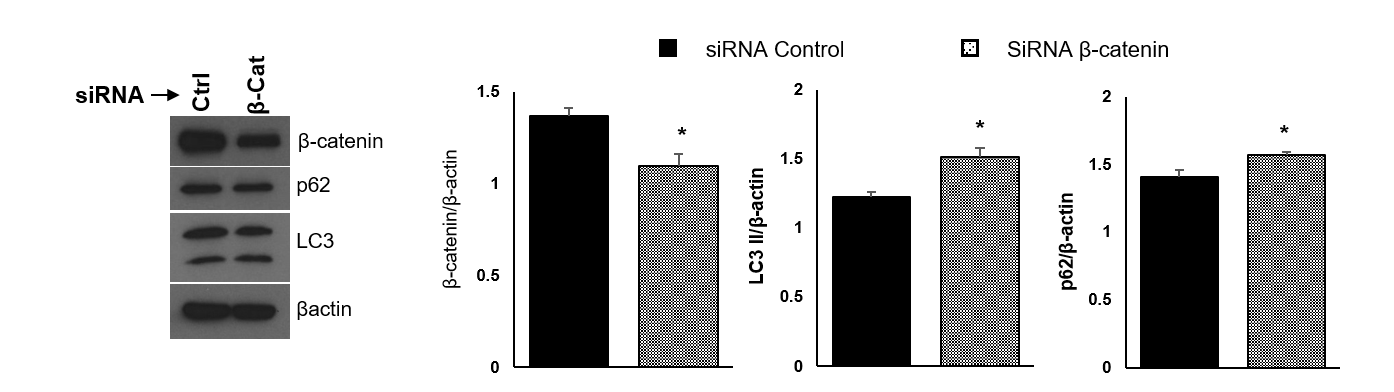

Supplement: S1 Fig — Western blot analysis of LC3BII and p62 protein levels of Huh7 transiently transfected with a β-catenin (β-cat) or control (Ctrl) siRNA. (TIF) [file pone.0212538.s002.tif]

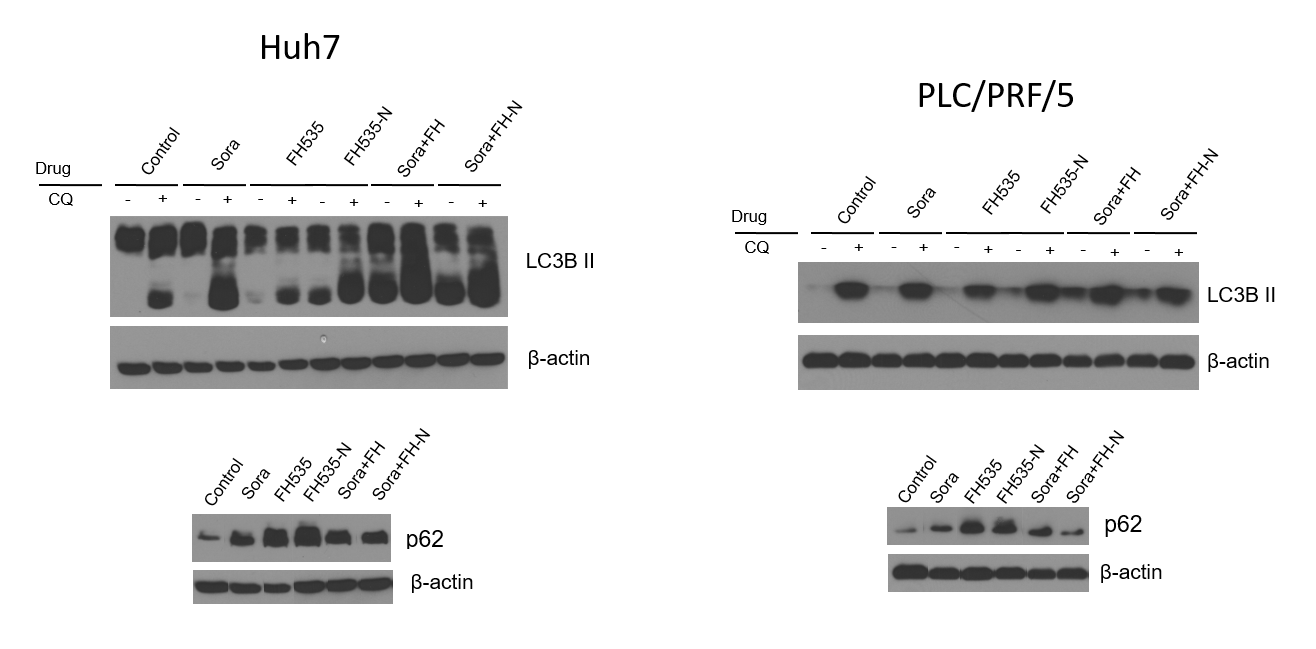

Supplement: S2 Fig — (TIF) [file pone.0212538.s003.tif]
